# The Georgia Medical Association

**Published:** 1871-05

**Authors:** 


### THE GEORGIA MEDICAL ASSOCIATION.

This body met at Americus, Ga., on the 12th ult. The Association was opened in due and regular form, but the proceedings will show that the manner of its organization, and character of its proceedings, may in the future develop some issues which may prove that the party, under the leadership of Dr. Logan, which succeeded in controlling and perverting it from the legitimate channels in which the body has so honorably and harmoniously moved in the past, may, like the boy, have drawn an elephant, which it may not be able to manage. As editors—being only individual members of the Association—we do not propose *now* to touch upon what may be justly considered as revolutionary acts by Dr. Logan and his party, which has, by unconstitutional means, succeeded in thus unhinging and destroying the character of the Association. This we will leave, for the present, to the profession, the honor and integrity of which is, in our opinion, so seriously jeopardized by the revolutionary acts already mentioned. The profession, of necessity, is the custodian of its own honor; and that it will defend and uphold it we do not for a moment question. A party victory, secured by means so utterly repugnant to the sentiments of every unbiased professional gentleman of the country, cannot be but momentary. A just appreciation of the true objects of the profession, and the rigid enforcement of the ethics in our Association will bring a reaction so grand and mighty that it will overwhelm in confusion and shame those who by unlawful and revolutionary procedures pervert and destroy the principles upon which the whole success and interests of these bodies are founded.

We desire, in this connection, to return our warm thanks for the kindnesses and courtesies shown us by our professional brethren of the beautiful little city of Americus. May their shadows never grow less! Our only regret is that, the citizens of their beautiful city should have been the witnesses of one of the most disgraceful medical (so-called) jerrymandering scenes ever enacted on the soil of Georgia. For the honor of the profession and the State, we ardently trust it will be the last disgraceful victory of error over right, to stain and wound the fair name and reputation of our profession.

